# Procedural-based category learning in patients with Parkinson's disease: impact of category number and category continuity

**DOI:** 10.3389/fnsys.2014.00014

**Published:** 2014-02-19

**Authors:** J. Vincent Filoteo, W. Todd Maddox

**Affiliations:** ^1^Veterans Administration San Diego Healthcare SystemSan Diego, CA, USA; ^2^Department of Psychiatry, University of CaliforniaSan Diego, CA, USA; ^3^Department of Psychology, University of TexasAustin, TX, USA; ^4^Institute for Neuroscience, University of TexasAustin, TX, USA

**Keywords:** Parkinson's disease, category learning, implicit processes, procedural learning, striatum, basal ganglia

## Abstract

Previously we found that Parkinson's disease (PD) patients are impaired in procedural-based category learning when category membership is defined by a nonlinear relationship between stimulus dimensions, but these same patients are normal when the rule is defined by a linear relationship (Maddox and Filoteo, 2001; Filoteo et al., 2005a,b). We suggested that PD patients' impairment was due to a deficit in recruiting “striatal units” to represent complex nonlinear rules. In the present study, we further examined the nature of PD patients' procedural-based deficit in two experiments designed to examine the impact of (1) the number of categories, and (2) category discontinuity on learning. Results indicated that PD patients were impaired only under discontinuous category conditions but were normal when the number of categories was increased from two to four. The lack of impairment in the four-category condition suggests normal integrity of striatal medium spiny cells involved in procedural-based category learning. In contrast, and consistent with our previous observation of a nonlinear deficit, the finding that PD patients were impaired in the discontinuous condition suggests that these patients are impaired when they have to associate perceptually distinct exemplars with the same category. Theoretically, this deficit might be related to dysfunctional communication among medium spiny neurons within the striatum, particularly given that these are cholinergic neurons and a cholinergic deficiency could underlie some of PD patients' cognitive impairment.

## Introduction

It is now widely accepted that there are multiple category learning systems (Ashby et al., 1998, 2010; Smith et al., 1998, 2012; Ashby and Maddox, 2005, 2011) and that different neural systems play different roles in these systems (Knowlton et al., 1994, 1996; Poldrack et al., 1999; Ashby and Ell, 2001; Filoteo et al., 2001a,b, 2005a,b; Patalano et al., 2001; Keri, 2003; Reber et al., 2003; Shohamy et al., 2004a,b; Maddox et al., 2005a,b; Cincotta and Seger, 2007; Nomura et al., 2007; Price et al., 2009; Waldschmidt and Ashby, 2011). One of the more interesting, and potentially important lines of research in this area is the study of how some categories can be acquired without conscious awareness. This phenomenon, often referred to as procedural-based category learning, occurs when participants learn complex categorization rules, and despite highly accurate learning, they are unable to describe explicitly why any given exemplar belongs to a specific category.

The behavioral mechanisms of procedural-based category learning have received much attention in several recent studies with normal individuals (Gluck et al., 2002; Maddox and Ashby, 2004; Ashby and Maddox, 2005, 2011; Ashby and O'Brien, 2005). These studies have demonstrated that this form of category learning has distinct operating characteristics that differentiate it from other types of category learning processes, such as explicit category learning (Ashby et al., 2002, 2003a,b; Maddox et al., 2003, 2004a,b; Maddox and Ing, 2005; Worthy et al., 2013). For example, the perceptual similarity among exemplars has to occur along a continuum within each category for normal procedural-based learning to occur, whereas this is not the case for explicit category learning (Maddox et al., 2005a,b, 2007). Similarly, the number of categories to be learned does not differentially impact long-run accuracy in procedural-based category learning, but increasing the number of categories impedes the learning of explicit category rules (Maddox et al., 2004a,b).

Much has also been learned about the underlying neurobiology of implicit or procedural-based category learning by the functional imaging of normal individuals or by studying patients with neurological disorders. For example, fMRI studies with normal participants have identified the striatum as an important brain region for procedural-based category learning (Filoteo et al., 2006; Cincotta and Seger, 2007; Nomura et al., 2007; Waldschmidt and Ashby, 2011) and other studies have implicated midbrain dopamine regions in some implicit category learning tasks (Aron et al., 2004). Past work with patients with striatal dysfunction has also implicated this brain region in implicit forms of category learning. Knowlton et al. (1996), for example, demonstrated that patients with Parkinson's disease (PD) are impaired in learning probabilistically determined categories, a finding that has received considerable support in the literature (Shohamy et al., 2004a,b). Importantly, other patient studies have indicated that brain structure associated with explicit memory (hippocampus and diencephalon) do not contribute to the same extent to implicit forms of category learning (Knowlton et al., 1994) or the long-term retention of procedural-based categories (Filoteo et al., 2001b).

**Figure 1:**
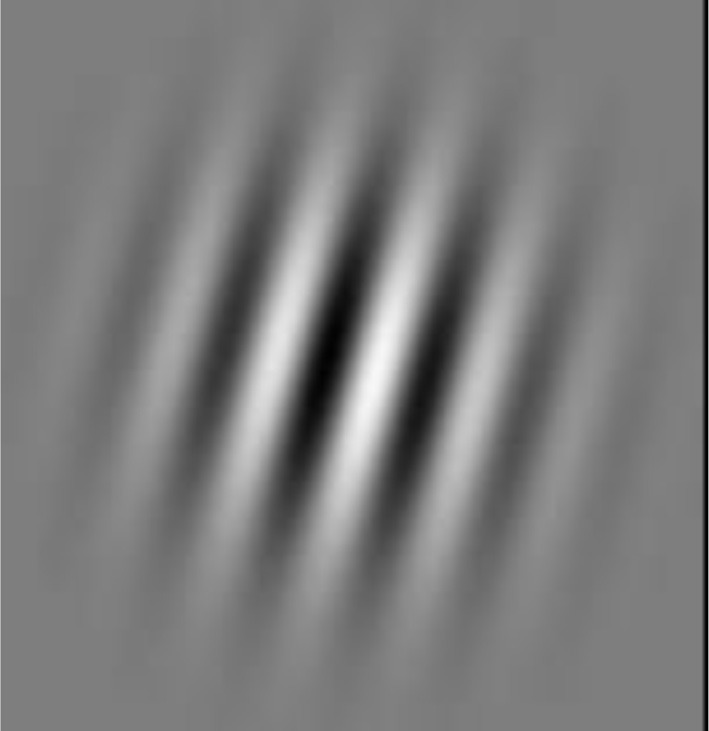
**Example of a Gabor stimulus used in Experiments 1 and 2**.

**Figure 2:**
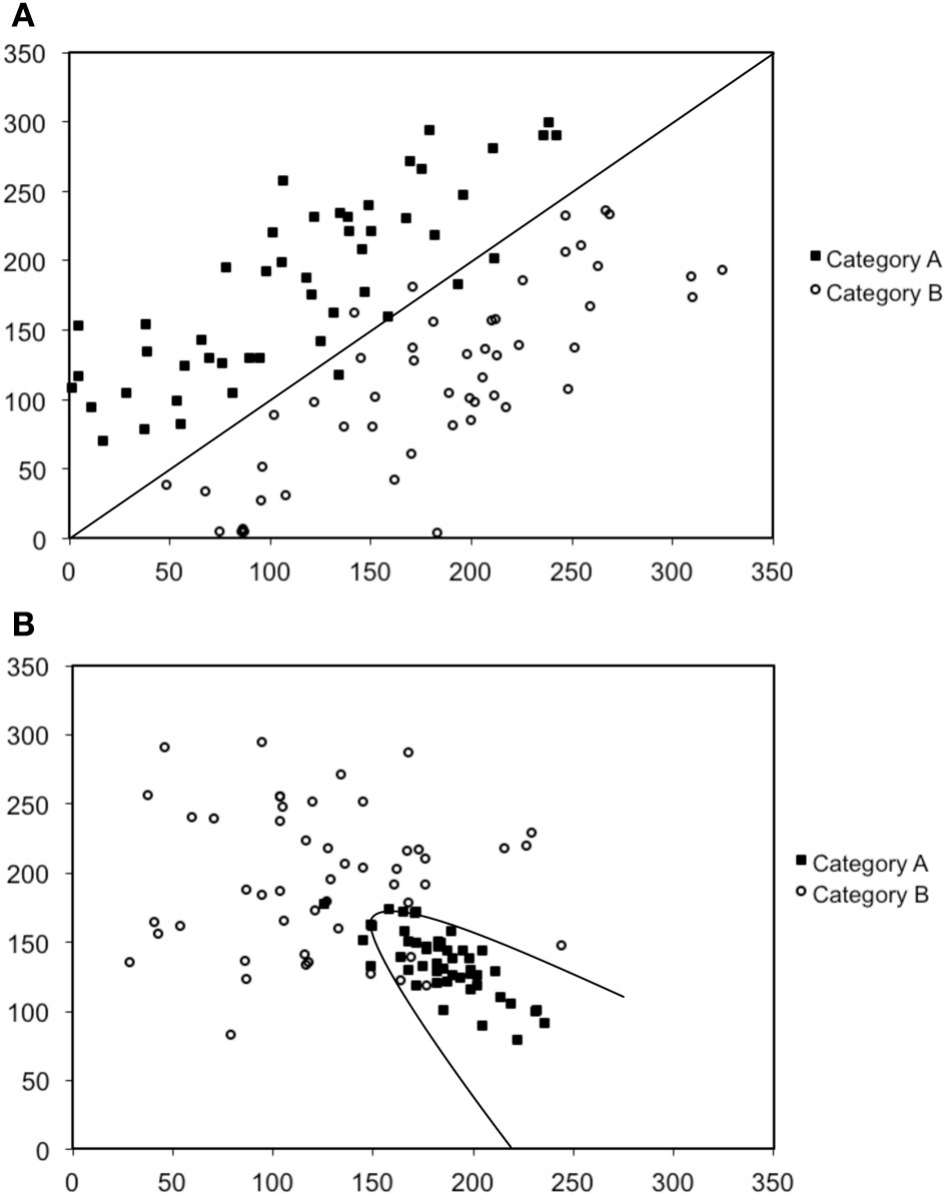
**Scatter-plots depicted examples of (A) a linear rule, and (B) a nonlinear rule.** Note that the scales are in arbitrary units.

In our work we have conducted a series of studies designed to further understand the nature of procedural-based category learning deficits in patients with PD, and by extension, the role of the striatum in this process (Maddox and Filoteo, 2001; Ashby et al., 2003b; Filoteo et al., 2005a). We have primarily used the perceptual categorization task (Ashby and Gott, 1988) in which participants view simple two-dimensional stimuli often consisting of a single line that varies in length and orientation (or a Gabor patch that varies in spatial frequency and orientation; see Figure 1) and are asked to categorize stimuli into one of two categories (Category A or B), and then immediately following a response, feedback is given. The rule that dictates category membership depends on the nature of the relationship between the two stimulus dimensions. Figures 2A,B provide examples in which the optimal rule is linear or nonlinear, respectively. This figure provides scatter plots of Category A and B stimuli where the x-axis represents the length of the line (in arbitrary units) and the y-axis represents the orientation of the line (in arbitrary units). Closed squares represent stimuli from Category A and open circles represent stimuli from Category B. Each individual stimulus has the length value on the x-axis and the orientation value on the y-axis. The linear rule depicted in Figure 2A is represented as a linear function and provides an optimal separation of the Category A and B stimuli, whereas the nonlinear rule in Figure 2B is represented as a quadratic function that provides an optimal separation of the Category A and B stimuli. A participant who would adopt the linear rule in Figure 2A or the nonlinear rule in Figure 2B would maximize long-run accuracy. Note that both rules are procedural-based category learning rules because it is very difficult to verbalize either the linear or nonlinear relationship between the two stimulus dimensions when they are not in the same perceptual units (e.g., length and orientation).

The results of our first study using this paradigm (Maddox and Filoteo, 2001) found that PD patients were impaired in learning a categorization rule that was based on a nonlinear relationship between lines that varied in length and orientation, whereas they were normal in learning a linear rule. Similarly, in our next study (Ashby et al., 2003a,b) we used a somewhat different task but again found that PD patients were normal in learning linear procedural-based rules. Finally, in a third study (Filoteo et al., 2005a,b) we again examined linear and nonlinear category learning and found that the patients were impaired in the nonlinear condition but not in the linear condition. Importantly, task difficulty could not explain these findings since the more difficult task (based on the accuracy of the control participants) was the linear task, on which PD patients were normal. This series of studies suggest that PD patients are impaired in procedural-based category learning, but only when the rule that dictates category membership is nonlinear.

A surface-level explanation of our findings is that PD results in deficits in learning nonlinear procedural based rules, but it does not impact linear rule learning. Unfortunately this explanation does not provide any insight into the possible mechanisms that might be driving these findings. To help interpret our past results we use the neurobiological and theoretical framework provided by the Striatal Pattern Classifier (SPC) model introduced by Ashby and colleagues (Ashby and Waldron, 1999). This model has been found to provide a good accounting of normal participants' response patterns in previous procedural-based category learning studies (e.g., Ashby and Waldron, 1999; Waldron and Ashby, 2001; Maddox et al., 2007; for applications to stimulus identification see Ashby et al., 2001; Maddox, 2001, 2002). The assumptions of this model are based on the neurobiology proposed to underlie the procedural-based category learning system in COVIS (Ashby et al., 2001). The SPC model, which is outlined in detail in Ashby and Waldron (1999), incorporates the knowledge of the many-to-one mapping of visual cortical cells onto cells in the striatum (Wilson, 1995). The model proposes hypothetical “striatal units” that are thought to represent the medium spiny cells in the striatum and provide a low-resolution map of the perceptual space. During procedural-based category learning the model assumes that these striatal units become associated with a category label and learn to associate a response with groups of cells in visual regions of cortex. It is important to be clear that the SPC is a computational model that is inspired by what is known about the neurobiology of the striatum. Because of this fact, the “striatal units” are hypothetical and could be interpreted within the language of some other computational model (e.g., as “prototypes” in a multiple prototype model).

**Figure 3:**
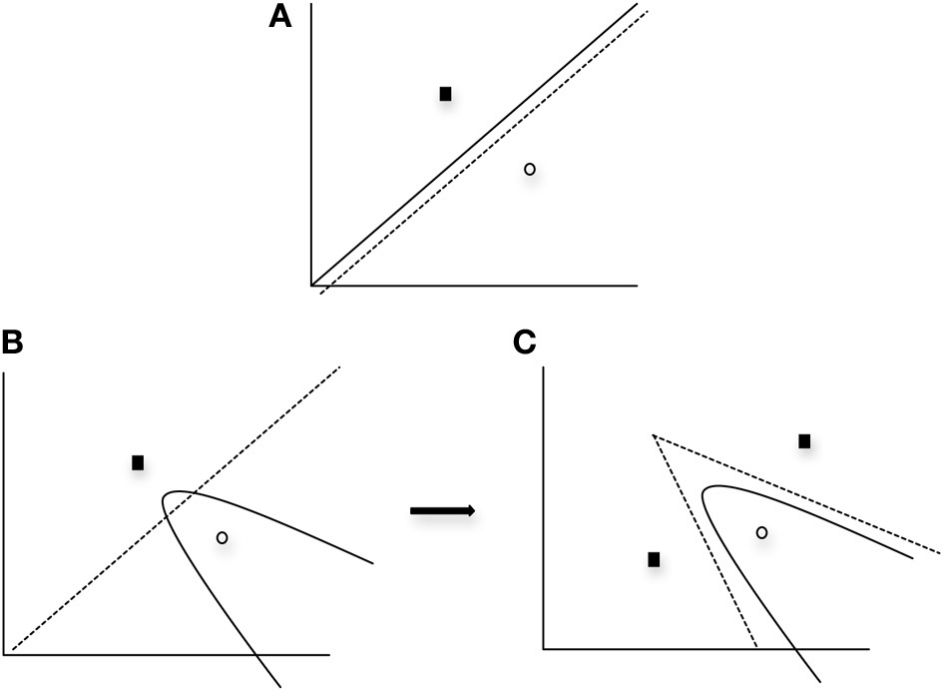
**Graphical example of the SPC modeling approach when (A) one unit per category is applied to a linear condition, (B) one unit per category is applied to a nonlinear condition, and (C) two units for one category and one unit for the other category is applied to a nonlinear condition.** Solid line and curves represent the “optimal” rule, whereas the dashed line and curves represent the partition between categories provided by the units. Note with the use of additional units for the nonlinear condition (panel **C**), the optimal quadratic bound (represented as the solid curve) is approximated by piecewise linear bounds. In all cases, black squares represent Category A units and open circles represent Category B units.

One important finding from the application of the SPC to data obtained from normal individuals (Ashby and Waldron, 1999) is that a greater number of striatal units are typically needed to represent a nonlinear rule as compared to a linear rule. The SPC is a minimum distance classifier. This is depicted in Figure 3A in which a linear rule is approximated by one striatal unit representing Category A (closed square) and another striatal unit representing Category B (open circle). In this case a minimum distance “bound” is learned. Note in Figure 3A that only a single unit per category is needed to approximate a linear rule. In contrast, Figure 3B provides a graphic representation of how striatal units might approximate a nonlinear rule. As can be seen in the first panel of Figure 3B, a single unit per category does not provide a good approximation of the optimal nonlinear rule. However, in Figure 3C the addition of a second striatal unit allows for a better approximation of the nonlinear rule via the piece-wise combination of two linear bounds so that two minimum distance bounds are learned. Thus, the SPC model argues that additional striatal units are needed to represent nonlinear rules.

This observation raised the interesting possibility that dysfunction in PD within these model-based “striatal units” might also reflect the pathological manifestations of PD within actual medium spiny neurons that compose the majority of cells within the striatum. These cells are the primary input nuclei in the striatum from the cortex and are part of the direct and indirect pathways within the basal ganglia. Medium spiny neurons are thought to be impacted in PD through the dysfunction of their dendritic spines due to deafferentation effects following the loss of dopamine cells within the pars compacta of the substantia nigra (Deutch et al., 2007), although this change might only be reflected functionally in the later stages of the disease (Zaja-Milatovic et al., 2005). Given the involvement of the medium spiny neurons in PD, one manner in which this disease could impact the proposed units is that the actual number of functional medium spiny neurons has diminished in these patients, and because of this, nonlinear categories that require a greater number of units can no longer be adequately represented. Thus, procedural-based learning conditions in which a greater number of units are required would always place PD patients at a disadvantage. We refer to this as the “number of units” hypothesis.

An alternative, but somewhat related possibility, is that the number of functional medium spiny neurons is normal in PD (at least early in the disease), but somehow these neurons are unable to communicate in a manner that would enable learning to occur when a greater number of striatal units is needed to represent the categories, such as under nonlinear conditions. That is, the number of functional medium spiny neurons is sufficient to support nonlinear category learning, but impairment in the ability of these neurons to communicate results in impaired learning. We refer to this as the “communication among units” hypothesis. This hypothesis was initially based on the observation from our previous studies that the learning of nonlinear rules requires that certain stimuli that are less perceptually similar have to be grouped into the same category, whereas certain stimuli that are more perceptually similar have to be grouped into different categories. Such communication among medium spiny neurons would be needed for the striatum to output a consistent message regarding that category to which a particular stimulus belongs. That is, unless there were some sort of co-activation among medium spiny neurons that processed the percept of a stimuli belonging to the same category, the output of these neurons would theoretically send a unique message to other structures eventually responsible for generating a response (e.g., the globus pallidus), and these structures would have to somehow resolve the fact that different medium spiny cells are signaling that their representation belongs to the same category. Note that this would only be the case when multiple units are required to represent a category, because theoretically, no such resolution would be required when only single medium spiny neurons (or medium spiny neurons within close proximity of one another) are needed to learn, such as under linear conditions.

An important question that needs to be addressed, however, is what could allow the medium spiny neurons to communicate. One possibility is that cholinergic interneurons that connect medium spiny neurons within the striatum enable such communication, and under conditions in which it would be theoretically beneficial for such cells to communicate (i.e., nonlinear conditions), striatal interneurons are involved in the learning process. These neurons often referred to as tonically active neurons (or TANs, for their tonic firing rate at rest) comprise only a small percentage of neurons in the striatum but recently have been implicated in processes important to procedural-based category learning (Ashby et al., 2007; Ashby and Crossley, 2011; Crossley et al., 2013). Specifically, most studies suggest that these interneurons modulate the input of cortical cells onto striatal medium spiny neurons by decreasing (or pausing) their activity when a rewarding stimulus is processed within the striatum, which allows for increased reinforcement learning (Apicella, 2002; Joshua et al., 2008; Aosaki et al., 2010). However, another method by which these interneurons result in learning could be by controlling the number of potential responses that are selected by the striatum (Stocco, 2012) and would be more consistent with a broader view of these interneurons in various aspects of learning (e.g., Apicella, 2007). This proposed process could provide an appropriate mechanism by which the striatum is able to link perceptually distinct stimuli to the same category response. This process is also consistent with other models of basal ganglia function that suggest a role of the striatum in response selection (Mink, 1996; Stocco, 2012), with reinforcement learning being one aspect of selecting a response (Bar-Gad et al., 2003; Redgrave et al., 2011) or linking networks within the striatum that are important for learning (Graybiel et al., 1994). Although the exact effects of interneurons on medium spiny cell function is not completely known and likely very complex (see Oldenburg and Ding, 2011), these cells do appear to play an important role in normal striatal functioning. Importantly, animal models of PD suggest that the reduction of dopaminergic projections to the striatum result in abnormal interneuron activity (Raz et al., 2001; Pisani et al., 2003; Bonsi et al., 2011).

**Figure 4:**
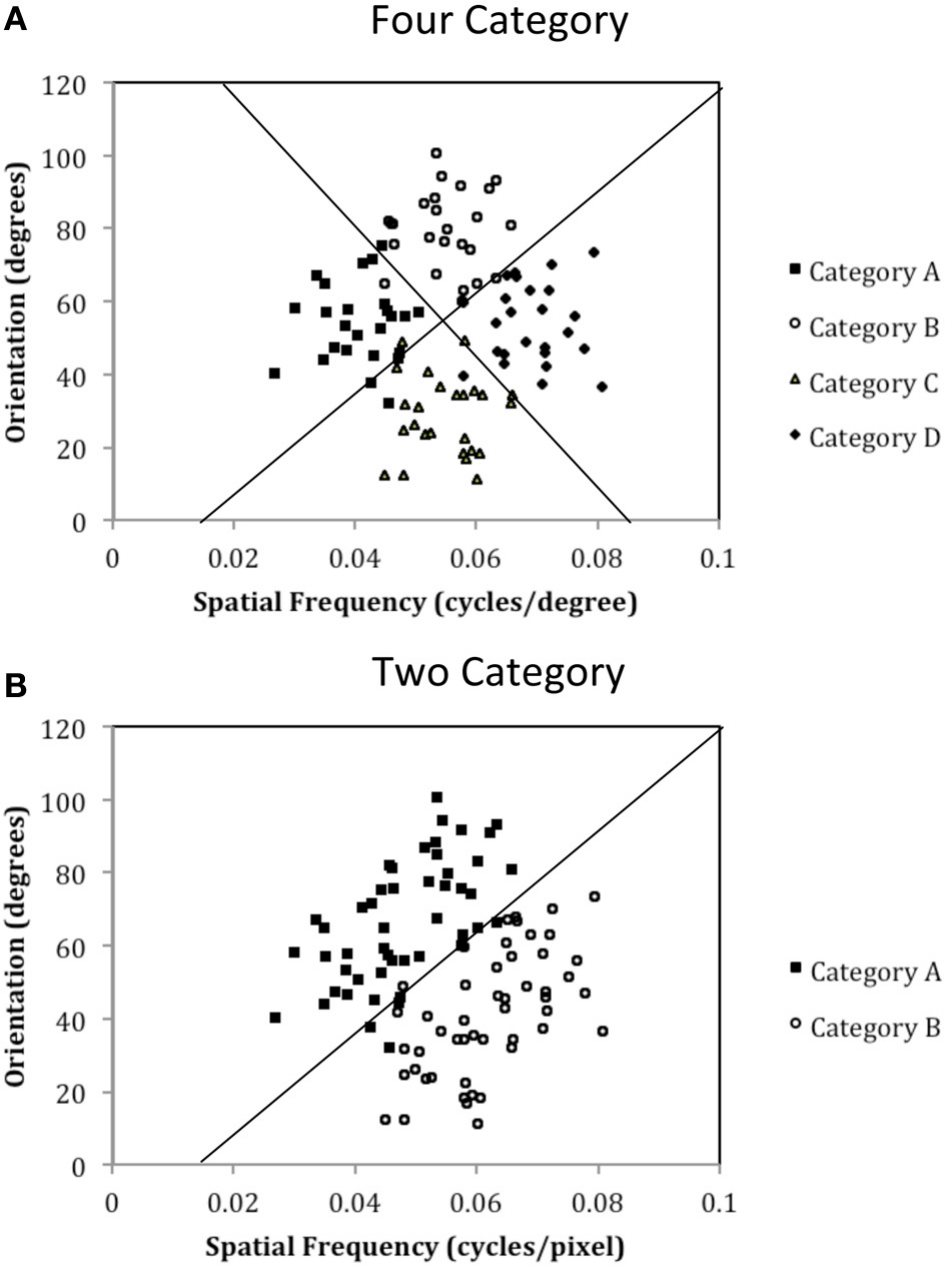
**Stimulus distributions for (A) the Four-Category and (B) Two-Category conditions in Experiment 1.** The solid line(s) represent the optimal rule(s).

The purpose of the current study is to examine both the “number of units” hypothesis and the “communication among units” hypothesis described above. Experiment 1 examined the ability of PD patients and normal controls (NC) to learn a procedural-based task in which there were either four categories (Four-Category condition) or two categories (Two-Category condition). Figure 4 displays the stimulus distributions for the Four- and Two-Category conditions. If PD results in a deficit in the number of hypothetical striatal units, then they should demonstrate greater impairment in the Four-Category condition as compared to the Two-Category condition. In contrast, Experiment 2 examined the “communication among units” hypothesis by determining the ability of PD patients and NC participants to learn categories that have either a discontinuous distribution of stimuli (Discontinuous condition) or a continuous distribution of stimuli (Continuous condition). Figure 6 displays the stimulus distributions for the Discontinuous and Continuous conditions. A finding that PD patients are impaired in the Discontinuous condition relative to the Continuous condition would provide theoretical support for the “communication among units” hypothesis of procedural-based category learning deficits in PD.

## General methods

### Participants

A total of 41 individuals participated in at least one of the two experiments: 20 PD patients and 21 NC participants. For the PD patients, 11 participated in at least one condition in both experiments and 9 participated in at least one condition in only one experiment. For the NC participants, 8 participated in at least one condition in both experiments and 12 participated in at least one condition in only one experiment. Participants were randomized to each experiment and in the case of those who participated in more than one experiment or in both conditions within an experiment, the order of administration of the experiments was randomized^1^. Participants were tested a minimum of 2 months apart between experiments or conditions. The specific numbers of individuals who participated in the two experiments are as follows. Experiment 1: Four-Category Condition, 12 PD patients (8 males and 4 females) and 12 NC participants (4 males and 8 females). Two-Category Condition, 11 PD patients (5 males and 6 females) and 11 NC participants (4 males and 7 females). Eight PD patients 8 NC participants were tested in both the Four-Category and Two-Category conditions. Experiment 2: Discontinuous Condition, 10 PD patients (6 males and 4 females) and 10 NC participants (4 males 6 females); Continuous Condition, 11 PD patients (8 males and 3 females) and 11 NC participants (5 males 6 females). Five PD patients and 7 NC participants were tested in both the Discontinuous and Continuous conditions.

The patients were recruited from Movement Disorder Clinics at UCSD and were diagnosed by a board-certified neurologist with subspecialty training in movement disorders. The diagnosis was based on UK Brain Bank Criteria (Hughes et al., 1992). PD patients were not included in the study if they scored above a cut-off of 11 on the Geriatric Depression Scale or if they scored below 130 on the Mattis Dementia Rating Scale (MDRS; Mattis, 1988). For Experiment 1, 14 patients were taking daily L-dopa medication, 8 were taking a dopamine receptor agonist, 5 were taking an MAO inhibitor, 5 were taking a COMT inhibitor as part of their L-dopa preparation, 5 were taking amantadine, and 1 was taking an anticholinergic. For Experiment 2, 14 patients were taking daily L-dopa medication, 8 were taking a dopamine receptor agonist, 3 were taking an MAO inhibitor, 6 were taking a COMT inhibitor as part of their L-dopa preparation, 5 were taking amantadine, and 1 was taking an anticholinergic.

Tables 1, 3 show the mean age, years of education, scores on the MDRS for the PD patients and NC participants who participated in Experiments 1 and 2, respectively, and the mean Hoehn and Yahr Rating Scale (HYRS; Hoehn and Yahr, 1967) score and the length of illness (LOI; years) for the PD patients. In both experiments, the PD and NC groups did not differ in age, education, scores on the MDRS, or gender distribution (all *p*'s > 0.05).

**Table 1:** **Demographic characteristics and Mattis Dementia Rating Scale Scores of the PD patients and NC participants in the Four-Category and Two-Category Conditions of Experiment 1**.

**FOUR-CATEGORY CONDITION**										
	**Age**		**Education**		**MDRS**		**HYRS**		**LOI**	
	***M***	***SD***	***M***	***SD***	***M***	***SD***	***M***	***SD***	***M***	***SD***
PD	67.1	7.6	16.5	1.9	139.7	3.1	2.0	0.5	7.3	4.6
NC	66.2	8.8	17.5	1.0	141.0	2.1	–	–	–	–
**TWO-CATEGORY CONDITION**										
	**Age**		**Education**		**DRS**		**HY**		**LOI**	
	***M***	***SD***	***M***	***SD***	***M***	***SD***	***M***	***SD***	***M***	***SD***
PD	64.2	7.9	16.3	2.0	140.5	2.5	2.1	0.5	6.3	3.6
NC	66.1	7.4	17.0	1.9	141.0	2.3	–	–	–	–

HY, Hoehn and Yahr Rating Scale score; LOI, Length of Illness.

### Stimuli and stimulus generation

In both experiments, the stimuli consisted of a single Gabor patch (see Figure 1) that varied in orientation and spatial frequency. The stimuli were computer generated and displayed on a 21′ monitor with 1360 × 1024 resolution. Each Gabor patch was generated using MATLAB routines from Brainard's (1997) Psychophysics Toolbox, and each stimulus was 7 cm in diameter, which subtended a visual angle of about 8.8° from a viewing distance of 45 cm.

Both experiments used the randomization technique of Ashby and Gott (1988). For each experiment, an equal number of Category A and Category B stimuli were generated by sampling randomly from two bivariate normal distributions. Each random sample (*x*_f_, *x*_o_) was converted to a stimulus by deriving the frequency, *f* = 0.0025 + (*x*_f_/5000) cycles per pixel, and orientation, *o* = 0.36*x*_o_ degrees. The scaling factors were chosen in an attempt to equate the salience of frequency and orientation based on our past experience with these stimuli. Each category distribution is specified by a mean and a variance on each dimension, and by a covariance between dimensions. For both category structures it was always the case that the covariance matrix for Category A was identical to the covariance matrix for Category B. The categories differed only in the location of their means.

The exact parameter values for the two experiments are listed in Tables 2, 4, and the category structures are displayed in Figures 4, 6. Figure 4A displays the category structures for the Four-Category condition in Experiment 1. Each filled square denotes the spatial frequency and spatial orientation of a Gabor pattern from Category A, each open circle denotes the spatial frequency and spatial orientation of a Gabor pattern from Category B, each closed diamond denotes the spatial frequency and spatial orientation of a Gabor pattern from Category C, and each closed triangle denotes the spatial frequency and spatial orientation of a Gabor pattern from Category D. Figure 4B displays the category structures for the Two-Category condition in Experiment 1, and Figures 6A,B display the Discontinuous and Continuous conditions in Experiment 2, respectively. For these figures, each filled square denotes the spatial frequency and spatial orientation of a Gabor pattern from Category A, while each unfilled circle denotes the spatial frequency and spatial orientation of a Gabor pattern from Category B. The solid line(s) in Figures 4, 6 denotes the location of the optimal decision bound(s). The use of the optimal bound in each of the four experiments maximizes long-run accuracy. Optimal accuracy in each condition was 95% given the categories overlapped to some extent, and thus were probabilistic.

**Table 2:** **Category distribution parameter values for Experiment 1**.

**FOUR-CATEGORY CONDITION**					
**Category**	***M*_s*f*_**	***M*_o_**	***SD*_s*f*_**	***SD*_*o*_**	**cov_*sf,o*_**
A	0.038	54.0	0.006	10.6	0
B	0.055	84.0	0.006	10.6	0
C	0.055	24.0	0.006	10.6	0
D	0.072	54.0	0.006	10.6	0
**TWO-CATEGORY CONDITION**					
**Category**	***M*_s*f*_**	***M*_o_**	***SD*_s*f*_**	***SD*_*o*_**	**cov_*sf,o*_**
A_1_	0.041	54.0	0.006	10.6	0
A_2_	0.055	79.5	0.006	10.6	0
B_2_	0.055	28.6	0.006	10.6	0
B_2_	0.069	54.0	0.006	10.6	0

**Table 3:** **Demographic characteristics and Mattis Dementia Rating Scale Scores of the PD patients and NC participants in the Discontinuous and Continuous Conditions in Experiment 2**.

**DISCONTINUOUS CONDITION**										
	**Age**		**Education**		**MDRS**		**HYRS**		**LOI**	
	***M***	***SD***	***M***	***SD***	***M***	***SD***	***M***	***SD***	***M***	***SD***
PD	65.4	6.9	16.0	1.8	138.5	3.3	2.0	0.2	6.8	2.9
NC	66.5	7.8	16.9	1.4	140.0	2.3	–	–	–	–
**CONTINUOUS CONDITION**										
	**Age**		**Education**		**MDRS**		**HY**		**LOI**	
	***M***	***SD***	***M***	***SD***	***M***	***SD***	***M***	***SD***	***M***	***SD***
PD	64.8	9.6	16.1	2.3	139.9	2.1	2.1	0.7	6.0	4.7
NC	66.6	7.2	16.3	2.5	141.0	2.5	–	–	–	–

HY, Hoehn and Yahr Rating Scale score; LOI, Length of Illness.

**Table 4:** **Category distribution parameter values for Experiment 2**.

**DISCONTINUOUS CONDITION**					
**Category**	***M*_s*f*_**	***M*_o_**	***SD*_s*f*_**	***SD*_*o*_**	**cov_*sf,o*_**
A_1_	0.051	31.7	0.002	3.6	0
A_2_	0.064	54.7	0.002	3.6	0
B_1_	0.058	20.2	0.002	3.6	0
B_2_	0.070	42.8	0.002	3.6	0
**CONTINUOUS CONDITION**					
**Category**	***M*_s*f*_**	***M*_o_**	***SD*_s*f*_**	***SD*_*o*_**	**cov_*sf,o*_**
A_1_	0.051	31.7	0.002	3.6	0
A_2_	0.055	39.2	0.002	3.6	0
B_1_	0.058	20.2	0.002	3.6	0
B_2_	0.062	27.7	0.002	3.6	0

### Experimental procedure

For the Four-Category and Two-Category conditions in Experiment 1, 600 trials were presented in 6 blocks of 100 trials. For the Discontinuous and Continuous conditions in Experiment 1, 400 trials were presented and were broken down into 5 blocks of 80 trials. At the start of each condition, the participants were told that they were involved in a study that examined their ability to categorize simple stimuli, that a series of stimuli would be presented, and that they would be asked to categorize each as a member of Category A B, C, or D for the Four-Category condition of Experiment 1, or Category A or B in the Two-Category condition of Experiment 1, and both conditions in Experiment 2. They were also told that at the beginning of the experiment they may feel as though they were guessing, but as the experiment progressed, their accuracy would likely increase. Participants indicated their categorization responses by pressing designated keys on the computer keyboard. For each trial in both experiments, the stimulus was presented until the participant's categorization response was made and feedback was presented immediately after the response for 1 s that consisted of either the word “wrong” if their response was incorrect or “correct” if their response was correct. Once feedback was given, the next trial was initiated 1 s later.

## Experiment 1: four-category vs. two-category conditions

Experiment 1 was designed to examine the “number of units” hypothesis. In the Four-Category condition, the participant must learn to assign each stimulus to one of four categories. Theoretically, each category is represented by a single striatal unit that is linked to the corresponding response (A, B, C, or D). As can be seen in Figure 4A, these categories are derived from four clusters of stimuli with different means and standard deviations (see Table 2). In the Two-Category condition, these same four clusters of stimuli are again used, but now there are only two categories given that Category A and B stimuli from the Four-Category condition are collapsed into Category A for the Two-Category condition, and Category C and D from the Four-Category condition are collapsed into Category B for the Two-Category condition. Thus, the exact stimuli are held constant across the two conditions, as is the nature of the stimuli, the timing of the task trials, and the nature of feedback. The only thing that varied is the number of categories.

As noted above, it was anticipated that if PD patients' deficits in learning nonlinear rules was due to such rules requiring a greater number of units to represent nonlinearity (see Figure 3) and there was a deficiency in the number of units in PD patients, the “number of units” hypothesis would predict that PD patients would be differentially impaired in the Four-Category condition as compared to the Two-Category condition.

## Results

**Figure 5:**
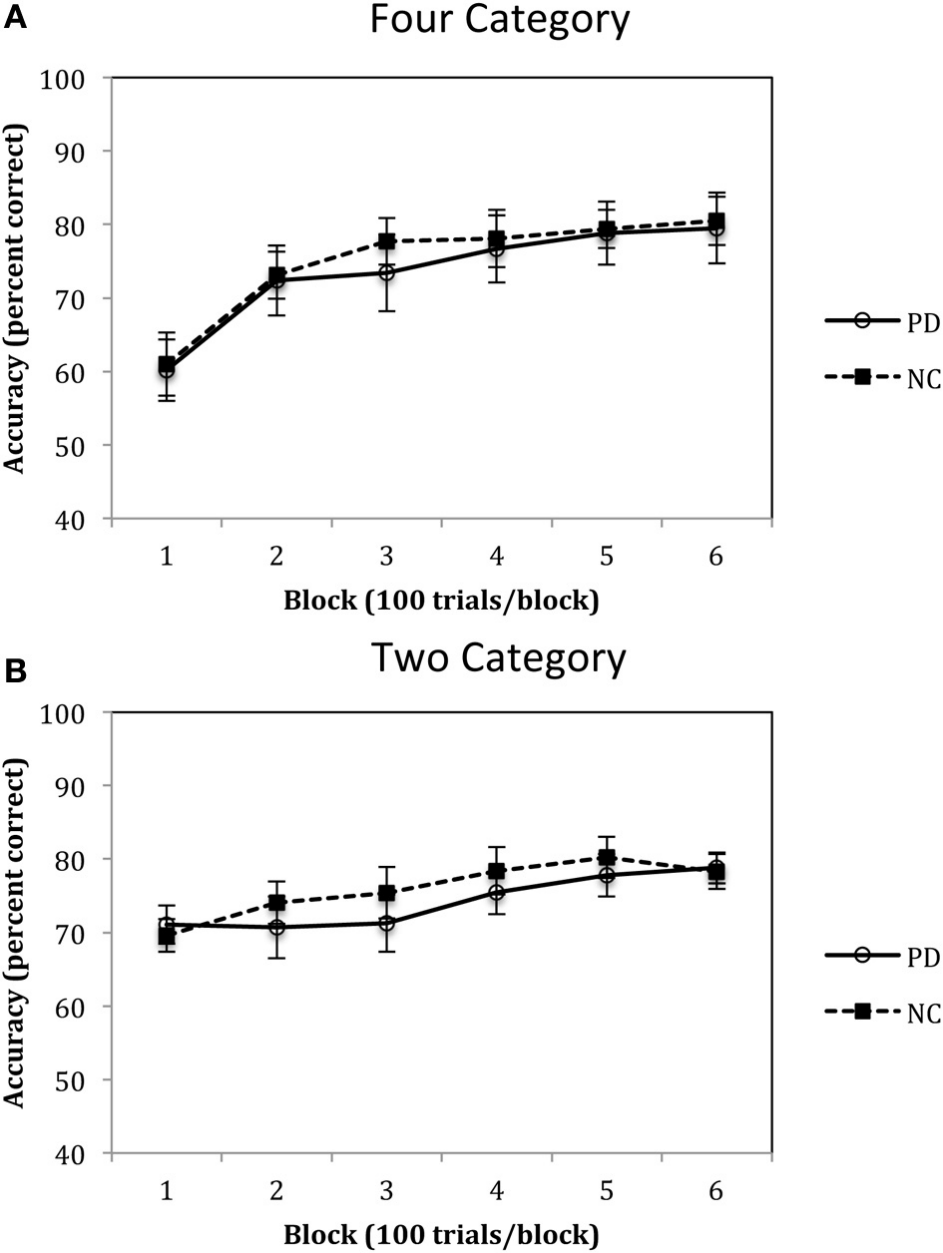
**Accuracy rates for PD patients and NC participants **(A)** Four Category and **(B)** Two Category conditions in Experiment 1.** (Error bars are in standard error of the mean).

Accuracy rates for the Four-Category condition of Experiment 1 are displayed in Figure 5A and were analyzed using a 2 (group: PD vs. NC) × 6 (blocks 1–6) mixed-design ANOVA. Results revealed a main effect of block, *F*_(5, 110)_ = 37.02, *p* < 0.001, η^2^_*p*_ = 0.63, with both PD and NC participants' performance improving across the trials. However, there was no main effect of group, *F*_(1, 22)_ = 0.08, *p* = 0.78, η^2^_*p*_ = 0.00, and no group by block interaction, *F*_(5, 110)_ = 0.35, *p* = 0.88, η^2^_*p*_ = 0.02. Accuracy rates for the Two-Category condition are displayed in Figure 5B and were analyzed using the same ANOVA design. Results indicated a main effect of block, *F*_(5, 110)_ = 11.88, *p* < 0.001, η^2^_*p*_ = 0.37, but no main effect of group, *F*_(1, 22)_ = 0.21, *p* = 0.65, η^2^_*p*_ = 0.00, and no group by block interaction, *F*_(5, 110)_ = 1.23, *p* = 0.30, η^2^_*p*_ = 0.06.

## Discussion

The results of Experiment 1 suggest that PD patients are not impaired when learning either four or two categories. As can be seen in Figures 4A,B, participants initially demonstrate a disadvantage in learning four categories as compared to two categories, but this is due to the fact that participants are initially guessing early in learning and chance responding in the four category condition is 25%, whereas in the two category condition it is 50%. However, as learning progresses, performance improves in both the Four- and Two-Category conditions and asymptotes at approximately 80% during the last block of trials. These findings are consistent with our previous work with healthy younger adults that showed little impact of category number on procedural-based category learning (Maddox et al., 2004a,b). The most important finding, however, is that there was no difference between PD patients and NC participants in the pattern and extent of learning in either the Four- or Two-Category conditions. If we can assume that normal learning in the Four-Category condition required a greater number of functional striatal units, then these findings do not support the “number of units” hypothesis.

## Experiment 2: discontinuous category vs. continuous category conditions

The purpose of Experiment 2 was to examine the impact of within-category discontinuity on procedural-based category learning in PD and NC participants. As noted above, we have found in three past studies that PD patients are not impaired in learning procedural-based category rules when the rule that dictates category membership is linear (Ashby et al., 2003a,b; Filoteo et al., 2005a,b) and our findings from Experiment 1 in this study provide further support for this observation. As we have argued above, one aspect of learning nonlinear rules is that participants must learn to categorize perceptual dissimilar stimuli into the same category so that they can activate the same response, and conversely, participants must learn not to categorize perceptually similar stimuli into the same category so that such stimuli can elicit a different response. This process is thought to occur through a response selection mechanism that is modulated by cholinergic interneurons within the striatum by inhibiting competing responses (e.g., Stocco, 2012). If there were a deficiency in communication among the medium spiny neurons within the striatum because of poor communication through the interneurons, then learning would be impaired. Again, we refer to this hypothesis as the “communication among units” hypothesis.

**Figure 6:**
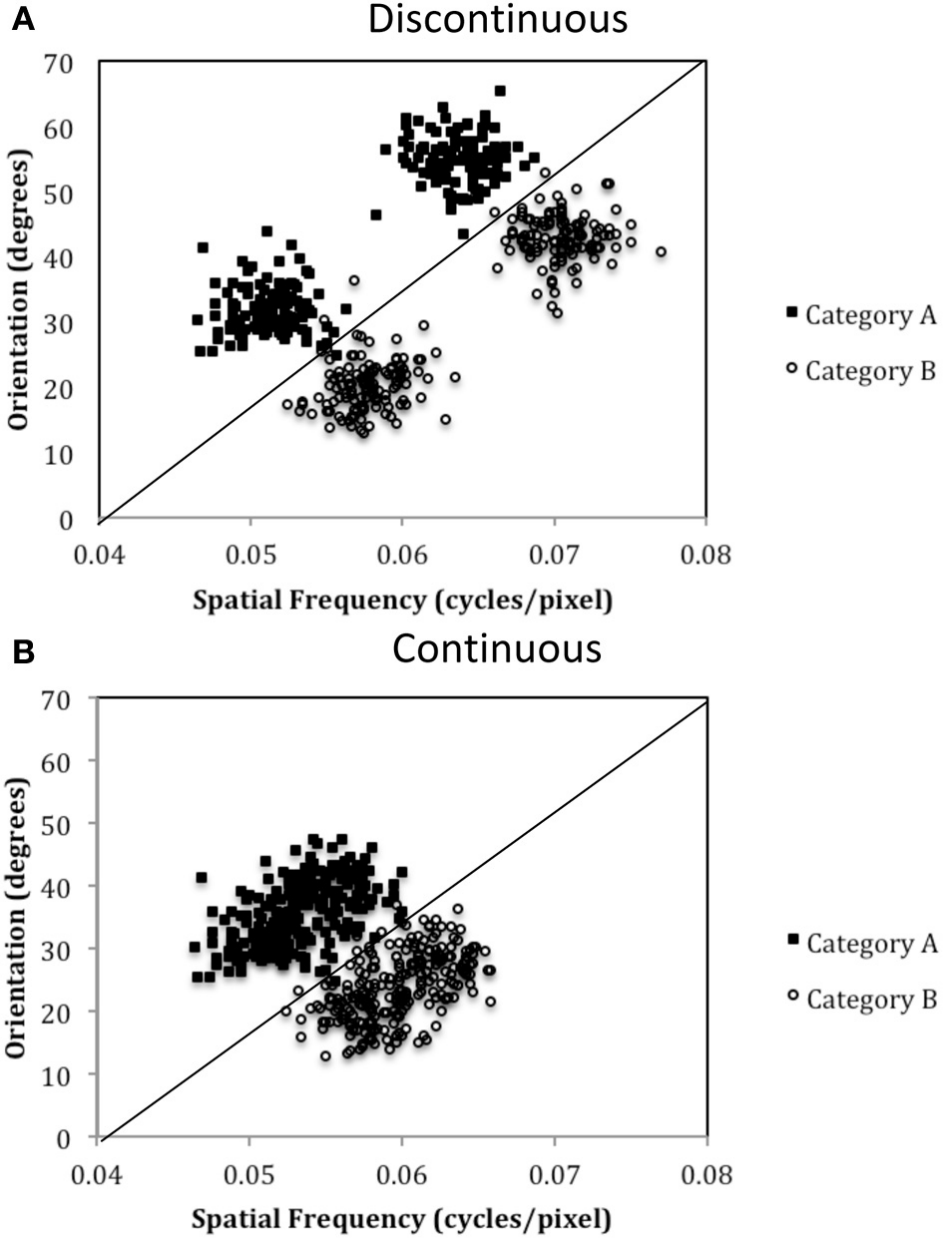
**Stimulus distributions for the (A) Discontinuous and (B) Continuous conditions in Experiment 2.** The solid line represents the optimal rule.

To test this hypothesis, we created a two-category condition in which a greater number of units would be needed to represent the stimuli within a single category but the rule was nevertheless linear. To do so, we created discontinuous categories by using two non-overlapping clusters within each category. As can be seen in Figure 6A, Category A stimuli (A_1_ and A_2_ clusters under Discontinuous condition in Table 4) compose two clusters as do Category B stimuli (B_1_ and B_2_ clusters under Discontinuous condition in Table 4). Importantly, stimuli from A_1_ and B_1_ are perceptually more similar than are A_1_ and A_2_ stimuli or B_1_ and B_2_. Thus, two important features of nonlinear rules are replicated: (1) perceptually dissimilar stimuli must be categorized together, and (2) more striatal units are needed to represent the categories; however, the rule is now linear. In contrast, in the Continuous condition, which served as the control condition, the categories were again composed of two clusters, but the clusters overlapped, which resulted in participants having to learn to categorize perceptually similar stimuli into the same category and a greater likelihood that only a single unit would be needed to represent the categories. If PD patients were differentially impaired in the Discontinuous relative to the Continuous condition, it would provide support for the “communication among units” hypothesis.

## Results

**Figure 7:**
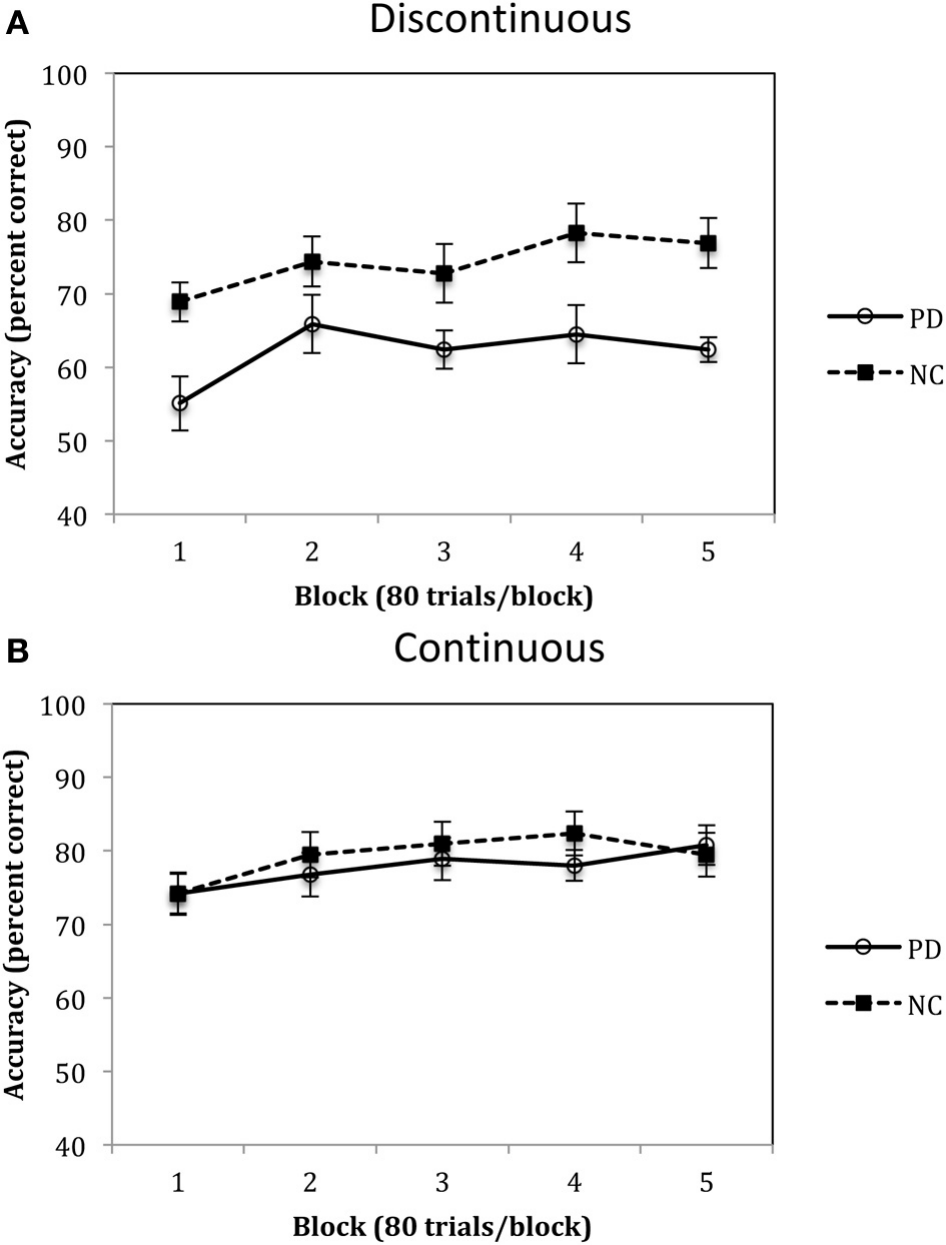
**Accuracy (percent correct) for PD patients and NC participants for the (A) Discontinuous and (B) Continuous conditions in Experiment 2.** (Error bars are in standard error of the mean).

Accuracy rates for Experiment 2 are depicted in Figure 7A and were analyzed using a 2 (group: PD vs. NC) × 5 (blocks 1–5) mixed-design ANOVA. Results of this analysis identified a main effect of group, *F*_(1, 18)_ = 6.68, *p* < 0.05, η^2^_*p*_ = 0.27, with PD patients performing worse than NC participants overall, and a main effect of block, *F*_(4, 72)_ = 11.06, *p* < 0.001, η^2^_*p*_ = 0.38, with both PD and NC participants' performances improving across the blocks. There was no group by block interaction, *F*_(4, 72)_ = 1.37, *p* = 0.25, η^2^_*p*_ = 0.07. Performances in the Continuous Condition are shown in Figure 7B and were examined using the same mixed-design ANOVA as for the Discontinuous Condition. Results of this analysis indicated that there was there was a main effect of block, *F*_(4, 80)_ = 4.37, *p* < 0.01, η^2^_*p*_ = 0.18, but no effect of group, *F*_(1, 20)_ = 0.21, η^2^_*p*_ = 0.01, and no group × block interaction, *F*_(4, 80)_ = 0.85, *p* = 0.50, η^2^_*p*_ = 0.04.

## Model based analyses

To further examine the results obtained in Experiment 2, we applied models to the final block of data separately from each participant (e.g., Estes, 1956; Maddox and Ashby, 1998; Smith and Minda, 1998; Maddox, 1999). The main class of model on which we focussed assumed that participants used an implicit procedural-based learning strategy—instantiated by applying the Ashby and Waldron's (1999) Striatal Pattern Classifier (SPC; see below for details). The model parameters were estimated using maximum likelihood (Ashby, 1992; Wickens, 1993) and the goodness-of-fit statistic was

AIC=2r−2lnL,

where *r* is the number of free parameters and *L* is the likelihood of the model given the data (Akaike, 1974; Takane and Shibayama, 1992). The AIC statistic penalizes a model for extra free parameters in such a way that the smaller the AIC, the closer a model is to the “true model,” regardless of the number of free parameters. Thus, to find the best model among a given set of competitors, one simply computes an AIC value for each model, and chooses the model associated with the smallest AIC value (for a discussion of the complexities of model comparisons see (Myung, 2000; Pitt et al., 2002).

The SPC model has been found to provide a good computational model of participants' responding in previous information-integration category learning studies (e.g., Ashby and Waldron, 1999; Waldron and Ashby, 2001; for applications to stimulus identification see Ashby et al., 2001; Maddox, 2001, 2002). In addition, the assumptions of this model are based on the neurobiology proposed to underlie the procedural-based system (Ashby et al., 2001). The SPC-1 assumes that there is one striatal unit for each category, and the SPC-2 assumes that there are two striatal units for each category. Both models assume a single noise parameter that estimates the variability associated with the participant's responding, with large variability estimates being associated with less deterministic responding and small variability estimates being associated with more deterministic responding. These models were developed to examine the possibility that participants in the discontinuous condition might learn to associate the separate, and distinct, sub-clusters of perceptually similar stimuli with the appropriate category. We hypothesized that if there was a deficit in communication and recruitment among the medium spiny neurons via dysfunction of the interneurons, then the SPC-1 model should be more likely to account for the pattern of PD patients' responding in the discontinuous condition, whereas an SPC-2 model would be more likely to account for the NC participants' responding. In contrast, there should be no difference between the groups in the continuous condition.

The results of the model applications supported our prediction in that only 1 out of 10 of the PD patients' data sets in the discontinuous condition were better fit by the SPC-2 model, whereas 4 out of 10 of the NC participants' data sets were best fit by the SPC-2 model. Furthermore, for both groups, those participants whose data were best fit by the SPC-2 model demonstrated better accuracy than those whose data were best fit by the SPC-1 model (69.8 vs. 58.8% for the PD group; 87.2 vs. 70.0% for the NC participants). In contrast, in the continuous condition, 0 out of 11 PD patients' data sets were better fit by the SPC-2 model, and only 1 of the 11 data sets from the NC participants was best fit by the SPC-2 model. Thus, in the discontinuous condition, the SPC model with a greater number of units was more likely to account for NC data sets, and this model was also associated with greater accuracy rates^2^.

## Discussion

The results from Experiment 2 indicated that, compared to NC participants, PD patients are impaired in procedural-based category learning when the categories are composed of discontinuous categories but are not impaired with continuous categories. Figure 7 also demonstrates the slight advantage NC participants have when learning continuous vs. discontinuous categories, a finding that we observed in our previous studies with healthy younger participants (Maddox et al., 2007). Of note, if the category clusters from the discontinuous condition (see Figure 6A) were from four different continuous categories, as opposed to two discontinuous categories, we would predict that PD patients would be normal.

Note this is the first study in which we found PD patients to be impaired in learning a linear procedural-based rule, arguing against the surface-level explanation that PD patients' deficits in category learning are simply due to the linearity of the rule. Rather, the present results support the hypothesis that PD patients are impaired in procedural-based category learning when there is a need for communication among striatal units, thereby supporting the “communication among units” hypothesis. We now turn to a discussion of the theoretical implications of our findings.

## Theoretical discussion

The main finding from the present set of experiments was that PD patients are impaired in learning discontinuous categories but are normal in learning continuous categories. In addition, these patients are not impaired when having to learn four categories. These findings provide initial support for our “communication among units” hypothesis. In contrast, the two groups did not differ in learning a procedural-based task with four categories, which does not support our “number of units” hypotheses.

The finding that PD patients are not impaired in learning either four- or two-category tasks suggests that the theoretical striatal units are functionally intact. This is consistent with the hypothesis that the medium spiny neurons were able to adequately represent multiple categories in our sample of PD patients. While it is known from PD animal models that the functional integrity of the medium spiny neurons can diminish in the absence of dopamine (Arbuthnott et al., 2000), post-mortem studies with actual PD patients suggest that structural changes to these neurons occurs only in later stages of the disease and may be the cause of motor complications secondary to dopaminergic treatment (i.e., dyskinesia). Specifically, Zaja-Milatovic et al. (2005) examined 9 PD patients post-mortem who had the disease a mean of 13 years and found that dendritic length of the medium spiny neurons was reduced in all striatal regions examined in PD patients relative to control post-mortem samples. The patients in our study tended to have the disease a mean of 6–7 years and were not displaying any complications of dopaminergic treatment (e.g., dyskinesia), so it is possible that the disease had not progressed in our patients to a point where the functioning of the medium spiny neurons had been impacted. This possibility raises the interesting question of whether PD patients with motor complications such as dyskinesia would be more likely to display deficits in the four-category condition given the possibility that their medium spiny neurons are less functional.

In contrast to the findings in Experiment 1, PD patients demonstrated a deficit in the Discontinuous condition in Experiment 2 but not in the Continuous condition. We have hypothesized that this is due to abnormal communication among the cholinergic interneurons in PD. We further hypothesized that normal interneuron communication is needed so that different medium spiny neurons that are processing perceptually dissimilar stimuli can resolve that they are representing stimuli that belong to the same category and are linked to the same response. This would only be required when there is a need for a greater number of the theoretical striatal units, such as when multiple units are needed to represent perceptually dissimilar exemplars from the same category (i.e., with discontinuous and nonlinear categories). Our assumption that striatal cholinergic interneurons are dysfunctional in PD is based on animal models that demonstrate increased activity of such neurons in the presence of reduced dopamine levels (Raz et al., 2001; Pisani et al., 2003; Bonsi et al., 2011). If this over activity of striatal interneurons is sufficient, improper signaling between medium spiny neurons would be likely to occur and the linking of perceptually dissimilar stimuli to the same response would be greatly compromised. This theoretical explanation is supported by other lines of research suggesting that the role of the basal ganglia, in general, and striatum, in particular, is to participate in response selection via the disinhibition of wanted responses and inhibition of unwanted responses (Mink, 1996; Stocco, 2012).

The possibility that cholinergic abnormality in PD underlies cognitive deficits in these patients is not new. However, the role of acetylcholine in PD cognition is not straightforward. On one hand there are previous studies indicating that medications that prevent the breakdown of acetylcholine (i.e., cholinesterase inhibitors) improve cognition in demented patients with PD (Emre et al., 2004; Bosboom et al., 2009; Possin et al., 2013). On the other hand we argue here that increased activity in cholinergic interneuron leads to a deficit in procedural-based category learning. Adding to this possible paradox are findings from a previous study where we demonstrated that impaired learning of a nonlinear procedural-based rule predicted future decline in global cognitive functioning in a group of nondemented PD patients (Filoteo et al., 2007). In addition, the fact that anticholinergic medications are often given to patients early in the course of the disease to improve motor symptoms by presumably reducing the over activity of the cholinergic interneurons also adds to the confusion as to how acetylcholine helps or hurts cognitive and motor functioning in PD. While we are unlikely to resolve these issues here, these possibilities raise the intriguing question of whether the administration of an anticholinergic would paradoxically improve nonlinear or discontinuous category learning in nondemented PD patients, or whether the use of a cholinesterase inhibitor would have any impact. These questions, and the general role of acetylcholine in PD cognition, certainly warrant further study.

It is important to note that the ideas tested in this paper are based on a hypothetical role of the function of cholinergic interneurons in the striatum and clearly represent an oversimplification of both the architecture and function of striatal medium spiny neurons and interneurons. At present, there is no neurobiological evidence to suggest that the specific role of these interneurons is to provide a conduit for which medium spiny neurons can link perceptually dissimilar stimuli to the same response. It may also be the case that the findings we report here are not due to such impairment but rather to some other mechanism, such as dysfunction in the output stage of response selection (e.g., Gurney et al., 2001). What is important is that we have further identified the experimental conditions under which PD patients demonstrate procedural-based category learning deficits, and that these data provide additional insights onto the mechanistic basis for some of our highly consistent previous results (Maddox and Filoteo, 2001; Ashby et al., 2003a,b; Filoteo et al., 2005a,b). In addition, the present work offers a potential computational understanding of the similarities between impaired nonlinear and discontinuous procedural-based category learning deficits in PD.

There are obviously several limitations to the present work. First, in regard to Experiment 1, it is possible that we did not tax the striatum sufficiently by the use of only four categories. It is possible that had we increased the number of categories we would have seen a deficit in the PD patients. As noted above, it is also possible that if we were to test patients in a more advanced stage of PD we would be more likely to see an impairment given the possibility that medium spiny neurons are only impacted in later stages of the disease (Zaja-Milatovic et al., 2005). Second, in regard to Experiment 2, there are several additional manipulations that could have been conducted to further examine the impact of discontinuity in PD patients' procedural-based category learning deficit. For example, in the present study we only examined one within category discontinuous separation and one between category separation. In other words, the within category cluster distance is fixed and so is the category (A vs. B) cluster separation. This issue could be examined parametrically to see what within and what between separations lead to a deficit. If, for example, we found that systematically increasing the between category separation decreases the magnitude of impairment in PD, this would further support the notion that perceptual similarity plays a key role in the observed deficit. Such manipulations are critical to further advance these theories. Third, in Experiment 2, the conditions did not only differ in terms of category continuity but also in terms of within-category range (i.e., how much of the stimulus space was occupied by category exemplars), which also could have explained the findings. However, in a previous study with healthy participants (Maddox and Filoteo, 2011) we found that category discontinuity had a greater impact on learning than did within-category range, suggesting that the results from the present study are less likely related to the degree of within-category range. Nonetheless, it will be important for future studies to directly examine this issue in PD.

In summary, the present study tested two theories of PD patients' deficits in procedural-based category learning. Our results and conclusions, while highly tentative and theoretical, suggest that PD patients are primarily impaired when learning requires perceptually dissimilar stimuli to be grouped in the same category, which may be due to dysfunctional communication among striatal units secondary to faulty communication.

### Conflict of interest statement

The authors declare that the research was conducted in the absence of any commercial or financial relationships that could be construed as a potential conflict of interest.

## Footnotes

^1^Additional analyses were conducted to determine if the order of administration of either the experiments or conditions could account for the pattern of results reported below and it was determined that this was not the case.

^2^It should also be noted that we applied a number of hypothesis-testing models that assumed the individual used an explicit approach to learning the categories. Although a few data sets were best fit by this class of models, the actual fits were close to those of the SPC models. Given specific questions we posed with the modeling and the small sample size we felt it was more important to focus on contrasting the SPC-1 and SPC-2 models.
